# Genomic sequence analysis of a plant-associated *Photobacterium halotolerans* MELD1: from marine to terrestrial environment?

**DOI:** 10.1186/s40793-016-0177-3

**Published:** 2016-09-01

**Authors:** Dony Chacko Mathew, Shou-Chen Lo, Gincy Marina Mathew, Kung-Hao Chang, Chieh-Chen Huang

**Affiliations:** 1 Department of Lifesciences, National Chung Hsing University, 145 Xingda Road, Taichung, Taiwan; 2 School of Biosciences, Mar Athanasios College for Advanced Studies (MACFAST) Biocampus, Tiruvalla, Kerala India

**Keywords:** Mercury, *Mer* operon, Glycine-Betaine, ROS, Rhizosphere, Heavy metals, *Photobacterium halotolerans*

## Abstract

Mercury impacts the function and development of the central nervous system in both humans and wildlife by being a potent neurotoxin. Microbial bioremediation is an important means of remediation of mercury-contaminated soil. The rhizospheric *Photobacterium halotolerans* strain MELD1 was isolated from mercury and dioxin contaminated site from Tainan, Taiwan. It has been shown to reduce Hg^2+^ to Hg^0^. The 4,758,027 bp genome of *P. halotolerans* MELD1 has a G + C content of 50.88 % and contains 4198 protein-coding and 106 RNA genes. Genomic analysis revealed the presence of a number of interesting gene cluster that maybe involved in heavy metal resistance, rhizosphere competence and colonization of the host plant.

**Electronic supplementary material:**

The online version of this article (doi:10.1186/s40793-016-0177-3) contains supplementary material, which is available to authorized users.

## Introduction

Species of the *Photobacterium* genus are Gram-negative bacteria belonging to the family of *Vibrionaceae* [1] and has been known to be marine bacteria either pathogenic [2] or symbiotic to marine life [3]. *Photobacterium halotolerans* was first reported by Rivas et al. [4] which was isolated from saline lake located in Mallorca, Spain.

In our previous study we isolated *Photobacterium halotolerans* MELD1, a plant growth promoting gamma-proteobacterium that was isolated from the root of *Phragmites communis* Trin. Ohwi [5], a large perennial grass found in wetlands throughout temperate and tropical regions of the world. The key feature of MELD1 was found to be the presence of *mer* operon, consisting of mercury reductase gene (*mer*A) that helped in the conversion of Hg^+2^ to Hg^0^ [6]. It was noted that MELD1 was resistant to mercuric chloride concentration up to a concentration of 33 μg/ml. In the present study we describe the summary genome classification of *P. halotolerans* MELD1 along with its annotation for rhizosphere competence, plant growth promoting and heavy metal resistant genes.

## Organism information

### Classification and features

**Fig. 1:**
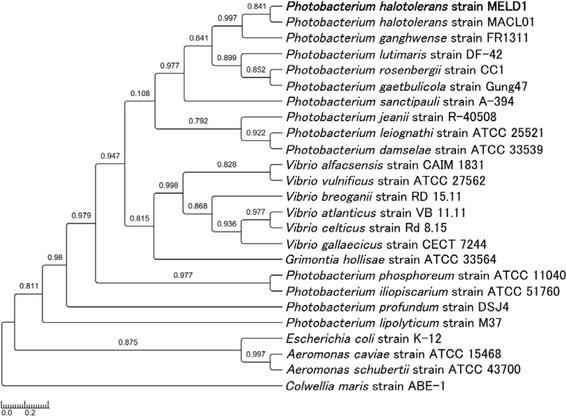
Phylogenetic tree based on 16S rRNA gene sequence of *Photobacterium halotolerans* MELD1 (highlighted) and other cultivated strains and clonal phenotypes within the phylum of *Gammaproteobacteria*. The 16S rRNA gene sequence were obtained from NCBI database and aligned with Clustal W and minimized with BioEdit. Phylogenetic tree file was generated by One Click phylogeny analysis on Methodes et Algorithmes pour la Bio-informatique Lirmm website (http://phylogeny.lirmm.fr/phylo_cgi/index.cgi). The scale bar represents a 0.03 % nucleotide sequence divergence

The genes encoding for the 16S rRNA were amplified by PCR using two universal primers, E8F and U1510R followed by BLAST against NCBI 16S rRNA sequences database. The 16S rRNA sequences of MELD1 and closely related strains were aligned by ClustalW and minimized with BioEdit (Tom Hall, Ibis Biosciences, Carlsbad, CA). The phylogenetic tree was generated by One Click phylogeny analysis on Methodes et Algorithmes pour la Bio-informatique Lirmm website [7] and exported by TreeGraph2 [8] (Fig. 1). Strain MELD1 demonstrated 99 % similarity to the *Photobacterium halotolerans* MACL01T as compared to other *Photobacterium* strains. Classification and general features of *P. halotolerans* MELD1 are shown in Table 1.

**Table 1:** Classification and general features of *Photobacterium halotolerans* MELD1 [31]

MIGS ID	Property	Term	Evidence code^a^
Classification		Domain *Bacteria*	TAS [32]
		Phylum *Proteobacteria*	TAS [33]
		Class *Gammaproteobacteria*	TAS [34]
		Order *Vibrionales*	TAS [35]
		Family *Vibrionaceae*	TAS [1]
		Genus *Photobacterium*	TAS [36]
		Species *Photobacterium halotolerans*	TAS [4]
		Type strain: MELD1	TAS [6]
	Gram stain	Negative	TAS [4]
	Cell shape	Rod shaped	TAS [4]
	Motility	Not reported	NAS
	Sporulation	Not reported	NAS
	Temperature range	4–37 °C	TAS [4]
	Optimum temperature	28 °C	TAS [6]
	pH range; Optimum	5–8.5, 7.4	IDA
	Carbon source	Glucose, Sucrose, L-arabinose	TAS [4]
MIGS-6	Habitat	Mercury contaminated soil	TAS [6]
MIGS-6.3	Salinity	6 % NaCl (*w/v*)	TAS [6]
MIGS-22	Oxygen requirement	Aerobic	IDA
MIGS-15	Biotic relationship	Plant Symbiont	TAS [6]
MIGS-14	Pathogenicity	Non pathogen	IDA
MIGS-4	Geographic location	Tainan, Taichung	TAS [6]
MIGS-5	Sample collection	2011	TAS [6]
MIGS-4.1	Latitude	23.3 N	TAS [6]
MIGS-4.2	Longitude	120.8E	TAS [6]
MIGS-4.4	Altitude	Not recorded	n/a

^a^Evidence codes – *TAS* Traceable Author Statement (i.e., a direct report exists in the literature), *IDA* Inferred from Direct Assay, *NAS* Non-traceable Author Statement (i.e., not directly observed for the living, isolated sample, but based on a generally accepted property for the species, or anecdotal evidence)

**Fig. 2:**
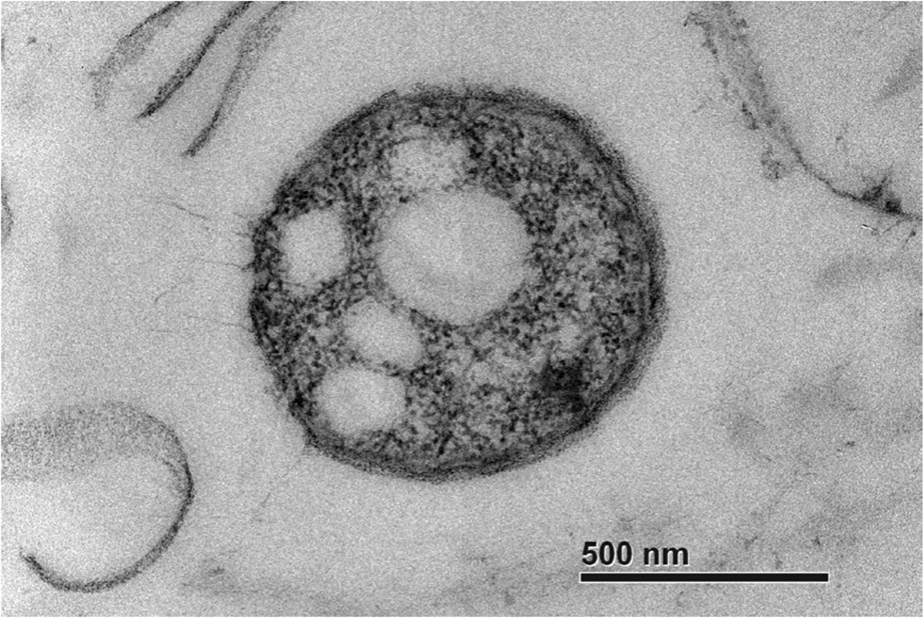
Transmission electron micrograph of *Photobacterium halotolerans* MELD1 cultured in Luria-Bertani medium (28 °C), using a Zeiss LEO 912 Energy-Filtering TEM. The scale bar represents 500 nm

Strain MELD1 is a Gram-negative bacterium, rod-shaped and motile by means of polar flagella. They are usually 2–4 μm in diameter (Fig. 2). *P. halotolerans* MELD1 could grow at 6 % NaCl as compared to *P. halotolerans* MACL01T which can grow at 8 % NaCl [4]. MELD1 was shown to utilize glucose, sucrose, maltose and α-D-Lactose as the sole carbon source.

## Genome sequencing information

### Genome project history

The genome project and the sequence were deposited in National Centre of Biotechnology Information [9]. MELD1 genome project consists of 62 contigs with a size of 4,758,037 bp, covering more than 97 % of the genome. A summary of the project information is showed in Table 2.

**Table 2:** Genome sequencing project information for *Photobacterium halotolerans* MELD1 genome

MIGS ID	Property	Term
MIGS 31	Finishing quality	Improved-high-quality draft
MIGS-28	Libraries used	Illumina Miseq paired end library
MIGS 29	Sequencing platforms	Illumina solexa technology
MIGS 31.2	Fold coverage	100×
MIGS 30	Assemblers	ABySS vs February 2014
MIGS 32	Gene calling method	FgenesB, GeneMarks+, Prodigal
	Locus Tag	KY46
	Genbank ID	JWYV00000000.1
	Genbank Date of Release	April 17, 2015
	GOLD ID	Go0106328
	BIOPROJECT	PRJNA260129
MIGS 13	Source Material Identifier	SAMN03263086
	Project relevance	Environmental, Bioremediation

### Growth conditions and genomic DNA preparation

*P. halotolerans* MELD1 was grown in Luria-Bertani medium under aerobic conditions at 28°C [4]. The genomic DNA was extracted by WelPrep DNA kit (Welgene Biotech, Cat No.D001). The size, purity and DNA concentration was measured by running pulse field gel electrophoresis, ratio of absorbance values at OD 260/280 in the range 1.8 ~ 2.0, and quantity ratio by Qubit versus NanoDrop over 0.7.

### Genome sequence and assembly

DNA was sequenced using Illumina Solexa technology. Ten microgram of total DNA was sonicated by Misonix 3000 sonicator to the size ranging from 400 to 500 bp. The genome size was estimated prior to assembly using Bioanalyzer DNA 1000 chip (Agilent Technologies). One microgram sonicated DNA was end-repaired, A-tailed and adaptor-ligated following the Illumina Trueseq DNA preparation protocol.

### Genome annotation

ConDeTri [10] was implemented to trim or remove the reads according to the quality score and the cleaned and filtered nuclear reads were assembled *de novo* using Abyss [11]. The gene functions were annotated using NCBI Prokaryotic Genome Annotation Pipeline, which uses has automatic annotation pipeline that combines *ab initio* gene prediction algorithms with homology based methods.

## Genome properties

**Fig. 3:**
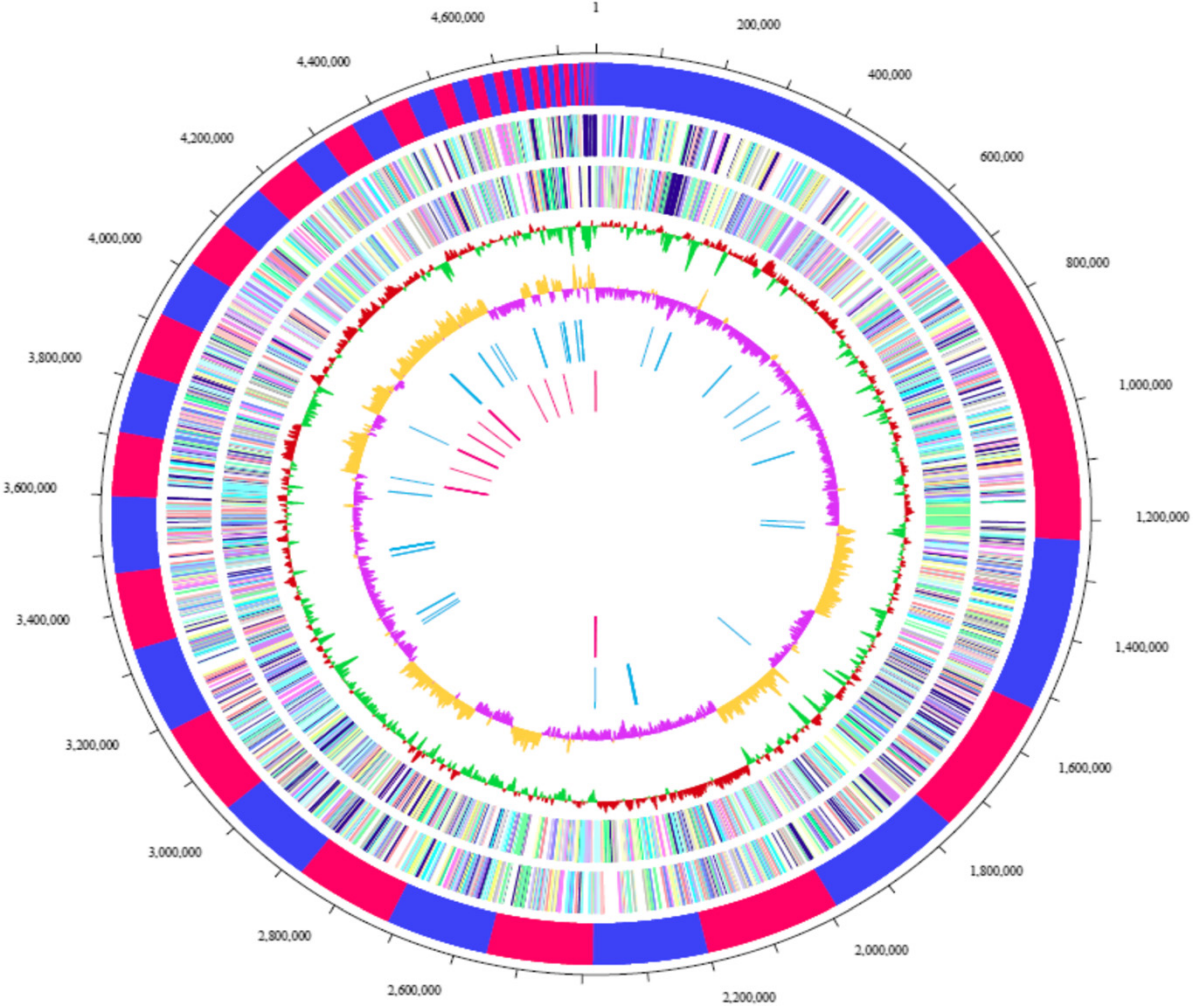
Schematic representation of circular replicon in *Photobacterium halotolerans* MELD1. The scale outside of the genomic map indicates genomic location (in kb). The bars in the outermost circle show the position of the scaffold delimitations for MELD1, represented in red and blue. The second and third circles from the outside depict the sense and antisense strands respectively. The fourth and fifth circle from outside represents the GC content (red and green) and GC skew values (pink and yellow) calculated using a window size of 1 kb. The innermost and second circle from the center represents tRNA in blue and rRNA in red

MELD1 genome contained 62 contigs with a size of 4,758,037 bp. The G + C content was 50.90 % (Fig. 3 and Table 3). Of the total 4382 genes, 4176 are protein-coding genes and 105 are RNA genes. The classification of genes based on COG functions is shown in Table 4.

**Table 3:** Genomic statistics for *Photobacterium halotolerans* MELD1

Attribute	Value	% of Total^a^
Genome Size (bp)	4,758,027	100.00
G + C content (bp)	2,420,749	50.88
DNA coding (bp)	4,054,779	85.22
Number of scaffolds	57	
Total genes	4382	100.00
RNA genes	106	2.43
Pseudogenes	65	1.49
Protein-coding genes	4198	96.09
Genes assigned to COGs	3509	80.32
Genes assigned Pfam domain	3650	83.54
Genes with signal peptides	407	9.32
Genes with transmembrane helices	1000	22.89
CRISPR repeats	2	

^a)^The total is based on either the size of the genome in base pairs or the total number of protein coding genes

**Table 4:** Number of genes associated with the general COG functional categories

Code	Value	% age^a^	Description
J	282	7.09	Translation, ribosomal structure and biogenesis
A	1	0.03	RNA processing and modification
K	310	7.79	Transcription
L	138	3.47	Replication, recombination and repair
B	1	0.03	Chromatin structure and dynamics
D	49	1.23	Cell cycle control, mitosis and meiosis
Y	0	0	Nuclear structure
V	101	2.54	Defense mechanisms
T	241	6.06	Signal transduction mechanisms
M	244	6.14	Cell wall/membrane biogenesis
N	144	3.62	Cell motility
Z	0	0	Cytoskeleton
W	38	0.96	Extracellular structures
U	93	2.34	Intracellular trafficking and secretion
O	185	4.65	Posttranslational modification, protein turnover, chaperones
X	65	1.63	Mobilome: prophages, transposons
C	208	5.23	Energy production and conversion
G	239	6.01	Carbohydrate transport and metabolism
E	326	8.2	Amino acid transport and metabolism
F	94	2.36	Nucleotide transport and metabolism
H	194	4.88	Coenzyme transport and metabolism
I	139	3.5	Lipid transport and metabolism
P	199	5	Inorganic ion transport and metabolism
Q	98	2.46	Secondary metabolites biosynthesis, transport and catabolism
R	332	8.35	General function prediction only
S	256	6.44	Function unknown
-	0	0	Not in COGs

^a^The total is based on the total number of protein coding genes in the genome

## Insights from the genome sequence

**Fig. 4:**
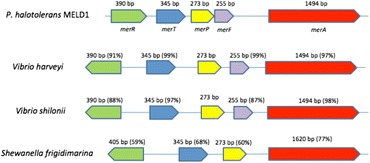
Comparative representation of *mer* operon of *Photobacterium halotolerans* MELD1 to *Vibrio* species and *Shewanella frigidimarina*

*Photobacterium halotolerans* MELD1 has been isolated from the rhizosphere of *Phragmites communis* Trin., a plant found growing in mercury and dioxin contaminated land. In our previous study, we had demonstrated the presence of *mer* operon, which helped in the conversion of Hg^+2^ to Hg^0^. The *Mer* operon of MELD1 was compared to the most similar Gram-negative bacteria in the NCBI database. It was observed the genes *merR*, *merT*, *merF*, *merP* and *merA* had varying degree of similarity compared to *Vibrio**shilloni,**Vibrio harveyi* [12] and *Shewanella frigidimarina* [13] as shown in Fig. 4.

Since *P. halotolerans* MELD1 was isolated from a plant growing in heavy metal environment, the bacteria had genes responsible for it to be a rhizosphere or endophytic bacteria and genes responsible for heavy metal resistance. MELD1 encodes genes responsible for rhizosphere competence like Siderophore, Chemotaxis, Quorum sensing, Glycine-Betaine, Tyrosine recombinase.

Many bacteria acquire iron from the environment by secreting small iron-binding molecules called siderophores. Bacteria have developed several mechanisms to compete for iron, an important element required for their growth. Siderophores are known to have an antagonistic effect by depriving iron from other microorganisms [14]. The presence of an effective iron uptake system can therefore contribute to protect the host plant against phytopathogens. Acquisition of iron is an important trait for rhizosphere competition [15]. Similar to other *Shigella* spp [16], *P. halotolerans* MELD1 is able to synthesize the siderophore aerobactin, it also encodes ferric siderophore transport proteins. Plant growth-promoting genes like nitrate reductase, *narL*, *ntrC* and phosphate transporters (*pst* and *pho*) are found to be in the MELD1 genome. Analysis of GC content of MELD1 genome (51 %) portrays that the gene that appear to have a GC content close to that of MELD1 genome, could have been integrated into MELD1 genome through evolution by the process of horizontal gene transfer (Additional file 1) [17–19].

Analysis of genome revealed that MELD1 has a number of gene reported to play a role in osmotolerance like glycine-betaine and ectoine. The genome analysis of MELD1 revealed genes involved in glycine betaine synthesis that help MELD1 to maintain osmotic balance in hyper saline environment. It was observed that MELD1 was able to grow at a salt concentration of up to 6 %. It carries an *ectABC* cluster responsible for synthesis and accumulation of ectoine (Additional file 2). Since we isolated MELD1 from a heavy metal contaminated environment, we identified genes responsible for heavy metal resistance to such as mercury, arsenic, copper, and tellurium as well gene’s responsible for antibiotic resistance and antimicrobial compound like phenazine (Additional file 3).

Plants utilize a variety of defense mechanisms against various pathogens, including the production of ROS, hydrogen peroxide [20, 21]. Prior to root colonization, MELD1 has to survive in an oxidative rhizosphere environment. The genome contains a number of genes that can play a role in detoxification of reactive oxygen species commonly found in bacteria’s growing in toxic environments. It includes peroxidase, superoxide dismutase, alkyl hydroperoxidase, hydroperoxidase DNA repair protein and universal stress protein. The MELD1 chromosome encodes two superoxide dismutases: SodA, an Mn superoxide dismutase, and SodB, a Fe superoxide dismutase. Acriflavine resistance protein B is another stress resistant gene induced upon by plant colonization, but it’s not triggered by oxidative stress. The product of this gene encodes a component of the AcrAB-TolC efflux pump that is important in toxic waste removal in bacteria and their expression increased during stress conditions [22, 23] (Additional file 4).

Adhension to the root in endophytic and rhizobacteria is mediated by cell surface structures such as polysaccharides, pili and adhesion [24]. It also carries a cluster of chemotaxis genes *cheY, cheW, cheA, cheR* and *cheX* (Additional file 5) and a cluster containing *flg* and *fli* genes responsible for flagella biosynthesis and motility (Additional file 6). It was also seen to possess the gene *xerD*, a site recombinase critical for the plant growth promoting rhizobacteria *Psuedomonas fluorescens* F113 to be an effective rhizosphere colonizer [25]. Quorum-sensing regulation gene in several strains of *Azospirillum lipoferum* [26] modulates functions related to rhizosphere competence and adaptation, such as siderophore synthesis, pectinase activity and indole acetic acid production [27]*.* MELD1 has quorum-sensing-regulatory genes like *luxR* and *luxU*, encodes AI-2 which is implicated in the regulation of biofilm formation and motility [28]. Some other genes involved for root adhesion including Hemaagglutinin [29, 30] are seemed to be responsible for the plant-microbe interaction as well as the twitching motility were observed in the MELD1 genome (Additional file 7).

## Conclusions

The 4.7 Mb draft genome of *Photobacterium halotolerans* MELD1, a strain having mercury reductase activity has been deposited at NCBI under the accession number JWYV00000000. The version described in this study is the first version, JWYV01000000. MELD1 also contained a cluster of gene’s responsible for heavy metal resistance, heavy metal efflux pumps, antimicrobial compounds, stress resistant, motility, and plant growth promoting genes, which all prove that they can function as a rhizosphere or an endophytic bacteria in a toxic environment. The detailed genome announcement can give insight into the adaption of a marine dwelling bacterium as a terrestrial dwelling endophytic or rhizosphere bacterium and in future might aid in the bioremediation of mercury. Further more extensive research need to be done using molecular techniques to establish horizontal gene transfer in MELD1 with donor species.

## Additional files

Additional file 1: Plant growth promoting genes. (DOCX 111 kb) Additional file 2: Genes responsible for osmotic stress resistance. (DOCX 50 kb) Additional file 3: Heavy metal and Antibiotic resistance genes. (DOCX 25 kb) Additional file 4: Genes responsible for detoxification. (DOCX 90 kb) Additional file 5: Genes responsible for rhizosphere competence. (DOCX 62 kb) Additional file 6: Gene responsible for motility. (DOCX 92 kb) Additional file 7: Genes responsible for secretion systems. (DOCX 69 kb)

## Abbreviations

ROS: Reactive oxygen species
