# Supplementary material for: Genomic sequence analysis of a plant-associated Photobacterium halotolerans MELD1: from marine to terrestrial environment?
Source: Stand Genomic Sci. 2016 Sep 1;11(1):56. doi: 10.1186/s40793-016-0177-3 (PMC5009661; doi:10.1186/s40793-016-0177-3)
Supplement: Additional file 1: — Plant growth promoting genes. (DOCX 111 kb) [file 40793_2016_177_MOESM1_ESM.docx]

|  | **Gene name** | **RefSeq** | **G+C content (%) *** |
| --- | --- | --- | --- |
| **Phosphate transporter (*pst*)** |  |  |  |
|  | *pstA* | WP_046218884.1 | 55.34 |
|  | *pstB* | WP_046218883.1 | 52.01 |
|  | *pstC* | WP_046218885.1 | 56.30 |
|  | *phoU* | WP_046218882.1 | 51.25 |
|  | *phoB* | WP_046218890.1 | 53.04 |
|  | *phoR* | WP_046218889.1 | 55.63 |
|  |  |  |  |
| **Phosphonate** |  |  |  |
|  | *phnA* | WP_046219031.1 | 47.66 |
|  | *phnT_2_* | WP_046221248.1 | 53.29 |
|  | *phnU_2_* | WP_046221249.1 | 53.60 |
|  | phosphonate ABC transporter substrate-binding protein | WP_046221247.1 | 52.56 |
| **Polyphosphate** |  |  |  |
|  | *ppx* | WP_046218888.1 | 55.26 |
|  | *Ppk_2_* | WP_046218881.1 | 50.24 |
|  |  |  |  |
| **Nitrogen fixation** |  |  |  |
|  | *napB* | WP_046221580.1 | 54.08 |
|  | *napD* | WP_046221581.1 | 52.38 |
|  | Nitrate/nitrite response regulator | WP_046221482.1 | 54.40 |
|  | Nitrate ABC transporter ATP binding protein | WP_046221610.1 | 56.60 |
|  | *ntrC* | WP_046218929.1 | 54.95 |
|  | Nitrate ABC transporter substrate binding protein | WP_046221612.1 | 56.63 |
|  | *amtB* | WP_046218929.1 | 55.44 |
|  | *glnG* | KKC98682 | 59.46 |
|  | *glnL* | WP_046221861.1 | 56.90 |
| **Siderophore** |  |  |  |
|  | *iutA* | WP_046220646.1 | 45.93 |
|  | *iucB* | WP_046220648.1 | 47.72 |
|  | *iucC* | WP_046220760.1 | 48.99 |
|  | *batA* | WP_046218632.1 | 55.68 |
|  | *fhuB* | WP_046221753.1 | 58.84 |
|  | *tonB* | WP_046221190.1 | 50.72 |
|  | *exbD* | WP_027251132.1 | 48.90 |

**Additional File 1.** Plant growth promoting genes.

*GC content of the selected genes was calculated using the Endmemo software (http//endmemo.com/bio/gc.php)
