# Supplementary material for: Genomic sequence analysis of a plant-associated Photobacterium halotolerans MELD1: from marine to terrestrial environment?
Source: Stand Genomic Sci. 2016 Sep 1;11(1):56. doi: 10.1186/s40793-016-0177-3 (PMC5009661; doi:10.1186/s40793-016-0177-3)
Supplement: Additional file 2: — Genes responsible for osmotic stress resistance. (DOCX 50 kb) [file 40793_2016_177_MOESM2_ESM.docx]

| **Product name** | **Gene symbol** | **Genbank Accession number** |
| --- | --- | --- |
|  |  |  |
| **Glycine-betaine** |  |  |
|  | *betI* | KKC98777 |
|  | *betB* | KKC98778 |
|  | *proX* | KKC98779 |
|  | *proW* | KKC98780 |
|  | *proV* | KKC98781 |
| **Ectoine** |  |  |
|  | *ectC* | KKD00912 |
|  | *ectB* | KKD00913 |
|  | *ectA* | KKD00914 |
|  |  |  |
|  |  |  |

**Additional File 2.** Genes responsible for osmotic stress resistance.
