# Supplementary material for: Genomic sequence analysis of a plant-associated Photobacterium halotolerans MELD1: from marine to terrestrial environment?
Source: Stand Genomic Sci. 2016 Sep 1;11(1):56. doi: 10.1186/s40793-016-0177-3 (PMC5009661; doi:10.1186/s40793-016-0177-3)
Supplement: Additional file 3: — Heavy metal and Antibiotic resistance genes. (DOCX 25 kb) [file 40793_2016_177_MOESM3_ESM.docx]

| **Product name** | **Gene symbol** | **GenBank accession number** | **GC content (%) *** |
| --- | --- | --- | --- |
| **Metal resistance** |  |  |  |
|  |  |  |  |
| **Mercury** |  |  |  |
|  | *merR* | KKC98119 | 43.76 |
|  | *merT* | KKC98120 | 48.85 |
|  | *merP* | KKC98121 | 46.01 |
|  | *merF* | KKC98122 | 45.73 |
|  | *merA* | KKC98123 | 47.16 |
|  |  |  |  |
| **Arsenic** |  |  |  |
|  | *arsB* | KKC98124 | 43.28 |
|  | *arsR* | KKC98125 | 44.83 |
| **Copper** |  |  |  |
|  | *copA* | KKC99043 | 56.47 |
|  | *cutC* | KKD01559 | 58.03 |
|  | Copper binding protein | KKD00728 | 51.49 |
|  | *Ctr1* | KKD00725 | 53.46 |
| **Tellurium** |  |  |  |
|  | *terD* | KKC99534 | 54.86 |
|  |  |  |  |
| **Extradiol dioxygenase** |  |  |  |
|  |  | KKD01366 | 52.75 |
| **Phenazine production** |  |  |  |
|  | *phzF* | KKD01616 | 49.82 |
| **Chloramphenicol resistance permease** | *Rad*D | KKC98668 | 58.33 |
|  |  |  |  |
| **Aromatic hydrocarbon degradation protein** |  | KKD01128 | 52.2 |
|  |  |  |  |
| **Antibiotic biosynthesis monooxygenase** |  | KKC99319  KKC98791  KKD01115 | 46.53  50.68  49.57 |
| **Antibiotic ABC transporter substrate binding protein** |  | KKD00764 | 49.09 |
| **Beta-lactamase** |  | KKD01206  KKD00757 | 54.2  48.91 |

*GC content of the selected genes was calculated using the Endmemo software (http//endmemo.com/bio/gc.php)

**Additional File 3.** Heavy metal and Antibiotic resistance genes.
