# Supplementary material for: Genomic sequence analysis of a plant-associated Photobacterium halotolerans MELD1: from marine to terrestrial environment?
Source: Stand Genomic Sci. 2016 Sep 1;11(1):56. doi: 10.1186/s40793-016-0177-3 (PMC5009661; doi:10.1186/s40793-016-0177-3)
Supplement: Additional file 4: — Genes responsible for detoxification. (DOCX 90 kb) [file 40793_2016_177_MOESM4_ESM.docx]

| **Product name** | **Gene symbol** | **GenBank accession number** |
| --- | --- | --- |
|  |  |  |
|  |  |  |
| **Glutathione Peroxidase** |  |  |
|  |  | KKD00732 |
|  |  | KKD00445 |
|  |  | KKD00539 |
|  |  | KKD00042 |
| **Peroxidase** |  |  |
|  |  | KKD01558 |
| **Peroxiredoxin** |  |  |
|  |  | KKC98890 |
|  |  | KKD00987 |
| **Superoxide dismutase** | *sod* |  |
|  |  | KKD01349 |
|  |  | KKD01342 |
| **Alkyl hydroperoxidase** |  |  |
|  |  | KKC99772 |
|  |  | KKD00257 |
| **Hydroperoxidase** |  | KKD00935 |
|  |  |  |
| **Glutaredoxin** |  |  |
|  |  | KKD01488 |
|  |  | KKD00988 |
|  |  | KKD00651 |
|  |  | KKC98551 |
|  |  | KKC98605 |
|  |  | KKC98117 |
|  |  | KKD01348 |
| **Acriflavin resistance protein** |  |  |
|  | *acrA* | KKC99572 |
|  | *acrB* | KKC99757 |
| **Metal binding heat shock protein** |  | KKC98690 |
|  | *hsp*15 | KKC98690 |
| **DNA repair protein** | *radA* | KKD01612 |
| **Recombination and repair protein** |  | KKD01581 |

**Additional File 4.** Genes responsible for detoxification.
