# Supplementary material for: Genomic sequence analysis of a plant-associated Photobacterium halotolerans MELD1: from marine to terrestrial environment?
Source: Stand Genomic Sci. 2016 Sep 1;11(1):56. doi: 10.1186/s40793-016-0177-3 (PMC5009661; doi:10.1186/s40793-016-0177-3)
Supplement: Additional file 5: — Genes responsible for rhizosphere competence. (DOCX 62 kb) [file 40793_2016_177_MOESM5_ESM.docx]

| **Product name** | **Gene symbol** | **GenBank accession number** |
| --- | --- | --- |
| **Chemotaxis** |  |  |
|  | *cheW* | KKD01142 |
|  | *cheY* | KKD01144 |
|  | *cheA* | KKD01145 |
|  | *cheR* | KKD01182 |
|  | *cheX* | KKC98095 |
| **Tyrosine recombinase** |  |  |
|  | *xerD* | KKD01691 |
|  | *xerC* | KKC98661 |
| ***lux* genes** |  |  |
|  | *luxR* | KKC98361 |
|  | *luxU* | KKD01332 |
|  |  |  |
|  |  |  |
|  |  |  |
|  |  |  |
|  |  |  |
|  |  |  |
|  |  |  |
|  |  |  |

**Additional File 5.** Genes responsible for rhizosphere competence.
