# Supplementary material for: Genomic sequence analysis of a plant-associated Photobacterium halotolerans MELD1: from marine to terrestrial environment?
Source: Stand Genomic Sci. 2016 Sep 1;11(1):56. doi: 10.1186/s40793-016-0177-3 (PMC5009661; doi:10.1186/s40793-016-0177-3)
Supplement: Additional file 6: — Gene responsible for motility. (DOCX 92 kb) [file 40793_2016_177_MOESM6_ESM.docx]

| **Product name** | **Gene symbol** | **GenBank accession number** |
| --- | --- | --- |
|  | *motY* | KKD01344 |
|  | *flhA* | KKD01149 |
|  | *flhB* | KKD01150 |
|  | *fliR* | KKD01151 |
|  | *fliQ* | KKD01152 |
|  | *fliP* | KKD01153 |
|  | *fliM* | KKD01156 |
|  | *fliL* | KKD01157 |
|  | *fliI* | KKD01159 |
|  | *fliH* | KKD01160 |
|  | *fliG* | KKD01161 |
|  | *fliF* | KKD01162 |
|  | *fliE* | KKD01163 |
|  | Flagellin B | KKD01169 |
|  | *flgL* | KKD01171 |
|  | *flgK* | KKD01172 |
|  | *flgJ* | KKD01174 |
|  | *flgH* | KKD01175 |
|  | *flgG* | KKD01176 |
|  | *flgF* | KKD01177 |
|  | *flgE* | KKD01178 |
|  | *flgD* | KKD01179 |
|  | *flgC* | KKD01180 |
|  | *flgB* | KKD01181 |
|  | *flgA* | KKD01184 |
|  | *flgM* | KKD01185 |
|  | *flgP* | KKD01188 |
|  | *flhF* | KKD01261 |
|  | *fliS* | KKD01262 |
|  | *flaI* | KKD01263 |
|  | *fliL* | KKD00140 |
|  | *fliI* | KKC99783 |
|  | *fliG* | KKC99784 |
|  | *fliE* | KKC99785 |
|  | *fliQ* | KKC99788 |
|  | *fliR* | KKC99789 |
|  | *flhB* | KKC99790 |
|  | *flhA* | KKC99791 |
|  | *fliS* | KKC99797 |
|  | *pomA* | KKC98969 |
|  | *motX* | KKC98109 |

**Additional File 6.** Gene responsible for motility.
