# Supplementary material for: Genomic sequence analysis of a plant-associated Photobacterium halotolerans MELD1: from marine to terrestrial environment?
Source: Stand Genomic Sci. 2016 Sep 1;11(1):56. doi: 10.1186/s40793-016-0177-3 (PMC5009661; doi:10.1186/s40793-016-0177-3)
Supplement: Additional file 7: — Genes responsible for secretion systems. (DOCX 69 kb) [file 40793_2016_177_MOESM7_ESM.docx]

| **Product name** | **Gene symbol** | **GenBank accession number** |
| --- | --- | --- |
| **Type IV** | *vgrG* | KKC97883 |
|  |  | KKC97889 |
|  |  | KKC97862 |
|  |  | KKC97845 |
|  |  |  |
| **Type II and III secretion systems proteins** |  | KKC99547 |
|  |  |  |
| **Type III effector** |  | KKD00397 |
|  |  |  |
|  |  |  |
| **Mannose sensitive Hemagglutinin (Type IVa)** |  |  |
|  | *mshA* | KKC99970 |
|  | *mshG* | KKC99972 |
|  | *mshM* | KKC99974 |
|  | *mshL* | KKC99975 |
|  | *mshJ* | KKC99977 |
|  | *mshI* | KKC99978 |
|  |  |  |
| **Pilus assembly protein** |  |  |
|  | *pilW* | KKD01796 |
|  | *pilZ* | KKD01613 |

**Additional File 7.** Genes responsible for secretion systems.
